# Trauma room requirements

**DOI:** 10.1007/s00068-025-02820-y

**Published:** 2025-03-18

**Authors:** Falco Hietbrink, Frank Hildebrand, Klemens Horst

**Affiliations:** 1 Department of Trauma Surgery, University Medical Center Utrecht, Utrecht, The Netherlands; 2 Department of Orthopaedics, Trauma and Reconstructive Surgery, RWTH Aachen University, Aachen, Germany

**Keywords:** Whitebook, Polytrauma, ESTE

## Abstract

Effective shock (or trauma) room management requires thorough preparation, staff competence, dedicated infrastructure and organised protocols. Shock rooms must be sufficiently equipped according to the hospital’s designated level of care. This chapter outlines essential aspects, including equipment and facilities, staff qualifications and composition, and communication practices. Key areas including initial assessment, diagnostic procedures, emergency interventions, and future aspects in the care of severely injured patients are addressed, along with emerging innovations in trauma care.

## Organisation

### Preparation

Preparation in trauma care begins with structured protocols, adequate communication with pre-hospital personnel (see Chap. 5), crew resource management, teamwork, and utilisation of facilities and resources. These elements should be established as the standard of care and practised regularly. Team members must be called immediately, preferably via an automated system, and must know their assigned roles and tasks.

### Team members

The resuscitation team may vary according to the country or level of care, but must include a sufficient number of trained specialists, including:

- Trauma team leader: The most experienced physician, preferably with surgical expertise, to oversee trauma management.
- Surgeons, anaesthesiologists, and specified nurses trained in trauma care.
- Radiology Personnel: To perform immediate diagnostic imaging.
- Specialists: Neurosurgeons or other experts are recommended to be present during resuscitation for severe trauma or available for consultation, depending on injury severity.

### Communication

Closed-loop communication, where input from team members is actively incorporated while the leader maintains an overview, is critical for coordinated care. Trauma team leaders must ensure constant information flow, and regular team training is mandatory to optimise communication and treatment skills.

### Facilities and resources

Shock rooms must provide:

- Diagnostic tools including X-ray equipment and ultrasound for eFAST.
- Resources for emergency procedures, including chest tubes, pelvic binders, C-clamp fixation, heaters, and rapid infusers.
- Supplies for massive transfusion protocols, including blood products. Familiarity with available blood products’ possibilities and limitations is essential for all team members.
- ABCDE-charts, handover checklists, and a visible clock for time tracking.

### Next steps

The chain of care must ensure:

- Intensive care unit (ICU) and operating room (OR) readiness.
- Early transport to facilities with adequate resources if required.
- Consultation with external specialists when necessary.

## Initial assessment and management requirements

### Training and workflow

All team members must be trained in the ABCDE approach, using recognised courses such as ATLS, ETC, or similar programmes, to ensure a common routine and language for processes, essential steps, and workflows. Throughput times should be as limited as possible to prioritise timely intervention. Regular trauma resuscitation practice is recommended to improve collaboration and reinforce the required team mindset.

Operating room team members must also undergo advanced trauma care training (e.g., ASSET ((Advanced Surgical Skills for Exposure in Trauma)), ATOM ((Advanced Trauma Operations Management)), DSTC ((Definitive Surgical Trauma Care)) (DATC ((Definitive Acute Trauma Care)) or DPNTC (Definitive Pre-Hospital and Trauma Care)), focusing on surgical procedures including clamshell thoracotomy, laparotomy, control of junctional bleeding, pelvic stabilisation and packing. These programmes develop critical skills in a team environment, emphasising communication and coordination for managing the most severely injured patients.

### Diagnostics

Timely diagnosis is critical for trauma patients. Shock rooms must be equipped with the necessary tools to detect life-threatening injuries, which must always be available. These include:

- Ultrasound and X-ray capabilities for the entire thorax and pelvis.
- Computed tomography (CT), which should ideally be located within the shock room to avoid risky handovers but must, at minimum, be nearby.
- Laboratory tests for instant data on basic vital parameters, such as blood gas analysis, with 24/7 accessibility.
- Visco-elastic point-of-care devices for coagulative status analysis, if available.
- Routine blood tests, which must be immediately transported to the laboratory.
- Interventional angiography to control bleeding of vessels near the trunk.
- Access to MRI, infrequently requested but available on a 24/7 basis when needed.

### Blood products

Trauma patients frequently require blood transfusions. Hospitals must provide immediate access to donor blood and its components. For those without in-house blood banks, reliable supply chains must be established. Pre-testing protocols should be in place to ensure timely availability, and facilities must have the capacity to conduct compatibility tests and antibody screening.

Protocols for massive transfusions must be readily available, regularly practised, and include appropriate checklists to streamline the process.

## Conclusion and needs for the future

Effective trauma room management relies on thorough preparation, skilled teams, and well-equipped facilities to provide optimal care for severely injured patients. Innovations such as hybrid operating rooms (ORs), which combine open surgery and endovascular procedures in a single setting, are transforming trauma care by streamlining logistics and reducing intervention times. To fully realise their potential, hybrid ORs must be integrated into daily practice and resuscitative algorithms, ensuring they become a standard component of trauma management.

Where hybrid ORs are not feasible, maintaining well-practiced routines in standard ORs remains essential to ensuring patient safety and minimising adverse events. Emerging technologies like 3D imaging further enhance trauma care by supporting navigated procedures and minimally invasive techniques, but these tools must complement—not replace—comprehensive trauma care strategies.

Looking ahead, standardisation of protocols, continued team training, and integration of advanced technologies will drive improvements in trauma room efficiency and outcomes. By combining preparation, expertise, and innovation, trauma systems can continue to save lives and reduce complications in the most critical moments of patient care.

## Data Availability

No datasets were generated or analysed during the current study.
